# Novel and traditional lipid-related biomarkers and their combinations in predicting coronary severity

**DOI:** 10.1038/s41598-017-00499-9

**Published:** 2017-03-23

**Authors:** Sha Li, Yuan-Lin Guo, Xi Zhao, Yan Zhang, Cheng-Gang Zhu, Na-Qiong Wu, Rui-Xia Xu, Ping Qing, Ying Gao, Xiao-Lin Li, Jing Sun, Geng Liu, Qian Dong, Jian-Jun Li

**Affiliations:** Division of Dyslipidemia, State Key Laboratory of Cardiovascular Disease, Fu Wai Hospital, National Center for Cardiovascular Diseases, Chinese Academy of Medical Sciences, Peking Union Medical College, BeiLiShi Road 167, Beijing, 100037 China

## Abstract

We investigated simultaneously traditional and novel lipid indices, alone or in combination, in predicting coronary severity assessed by Gensini score (GS) in 1605 non-lipid-lowering-drug-treated patients undergoing coronary angiography. Firstly, levels of triglycerides (TG), total cholesterol (TC), low density lipoprotein cholesterol (LDL-C), non high density lipoprotein cholesterol (non-HDL-C), apolipoprotein (apo) B, lipoprotein (a) [Lp(a)], proprotein convertase subtilisin/kexin type 9 (PCSK9), apoC3, small dense LDL (sdLDL) and large HDL were increased, while HDL-C and apoA1 levels were decreased as GS status (all p for trend <0.05). However, gender stratification analyses showed similar associations between lipids and GS in men but not in women. Secondly, multiple logistic regression analyses indicated that the 12 indices were predictive for high GS (≥24) but not for low GS (1–23) compared with normal coronary (GS = 0) except for TG (neither) and apoB (both). Finally, we found that interactions between two indices with mutually exclusive composition were positively associated with GS status except for couples of TC + apoC3, apoB/PCSK9/apoC3 + sdLDL-C. Concordant elevations in the two showed the highest predictive values for high GS (all p for trend <0.05). Therefore, lipid biomarkers were associated with coronary severity and their adverse changes in combination emerged greater risks in men but not in women.

## Introduction

Coronary artery disease (CAD) refers to atherosclerotic stenosis and/or myocardial ischemia, is increasing and life-threatening from young to old age^1, 2^. Along with advance in the knowledge of atherosclerosis, dyslipidemia is recognized as a major contributor for the development of CAD^3–5^. Therefore, understanding the interplay between circulating lipids and the risk of CAD is of great importance to clinical practice and public health.

Circulating total cholesterol (TC) or low density lipoprotein (LDL) cholesterol (LDL-C) is associated with the development of CAD in an independent and graded manner and fulfill the criteria for causality^3, 6^. Therapeutic reduction focusing on LDL-C is the cornerstone in disease management and cardiovascular benefit so far^6–8^. In fact, dyslipidemia is a complex condition with qualitative and/or quantitative disorders of lipids and lipoproteins, including abnormal levels such as elevated LDL-C or decreased high density lipoprotein (HDL) cholesterol (HDL-C), qualitative changes in particles, accumulation of remnant lipoproteins and postprandial hyperlipidemia^7^. There are good evidences that triglycerides (TG), HDL-C and lipoprotein (a) [Lp(a)] are related to the risk of CAD. Although the causality underlying these associations remains to be further studied^5, 9, 10^ and benefits of their level improvements are uncertain, their values in combination with LDL-C are gaining attention^11–13^.

Furthermore, traditional lipid measurements seem to have explained the major cardiovascular risk in diseased population, but it remains an unmet need for more diagnostic or therapeutic markers to evaluate CAD status and residual risk^14, 15^. To date, emerging biomarkers based on lipids have been identified, such as proprotein convertase subtilisin/kexin type 9 (PCSK9)^16, 17^, apolipoproteinC3 (apoC3)^18^, small dense LDL (sdLDL)^19^ and large HDL^20^. Hence, circulating lipid biomarkers, traditional or new emerging, alone or in combination, may be considered comprehensively in CAD risk evaluation.

However, the associations between these biomarkers and coronary atherosclerosis had been rarely studied. The purpose of the present study, therefore, was to investigate simultaneously the predictive values of traditional and new emerging lipid indices for coronary severity in a large cohort of Chinese non-lipid-lowering-drug-treated patients undergoing coronary angiography (CAG).

## Materials and Methods

### Study population

Our study complied with the Declaration of Helsinki and was approved by the hospital’s ethical review board (Fu Wai Hospital & National Center for Cardiovascular Diseases, Beijing, China). Informed written consents were obtained from all enrolled patients in this study.

To study the associations of lipid biomarkers with coronary severity, we consecutively enrolled the study subjects with none lipid-lowering-drug therapy and CAG being parts of the screening process in our division from October 2012 to May 2016. Specifically, the inclusion criteria were as follows: (1) no treatment history of statins and/or other lipid-lowering drugs at least 3 months prior to the admission; (2) completed information of anthropometric characteristics and standard cardiovascular risk factors^19–21^ and (3) definite evidence of coronary condition from CAG by our interventional physicians. Exclusion criteria were patients with acute coronary syndrome (ACS), serious heart failure or arrhythmia, psychiatric disorder, infectious or systematic inflammatory disease within 1 month, significant hematologic disorders, thyroid dysfunction, severe liver dysfunction (more than 3 times the upper limits of normal aspartate aminotransperase and/or alanine aminotrabsferase), renal insufficiency (creatinine >1.5 mg/dL) and malignant tumors. As a result, there were 1605 eligible patients in the analysis.

### Laboratory examinations

All patients underwent clinical examination and blood testing as our previous studies^19–21^. The concentrations of TC, TG, HDL-C, LDL-C, apoA1 and apoB were measured using an automatic biochemistry analyzer (Hitachi 7150, Tokyo, Japan). The TC, TG, HDL-C and LDL-C levels were measured using an enzymatic assay. ApoA1 and apoB levels were measured using a turbidimetric immunoassay. Hemoglobin A1c (HbA1c) was measured using Tosoh Automated Glycohemoglobin Analyzer (HLC-723G8, Tokyo, Japan). The concentrations of high-sensitive C-reactive protein (hs-CRP) were determined using immunoturbidimetry (Beckmann Assay 360, Bera, Calif., USA).

Plasma PCSK9 levels were measured by high-sensitivity, quantitative sandwich enzyme-linked immunosorbent assay using CircuLex ELISA kit. Plasma apoC3 levels were measured using the RayBio® ELISA kit, which was an *in vitro* enzyme linked immunosorbent assay for the quantitative measurement and employed an antibody specific for human apoC3. SdLDL-C analysis was performed electrophoretically by the Lipoprint LDL System and large HDL analysis was performed by the Lipoprint HDL System (Quantimetrix Corporation, Redondo Beach, CA, USA) according to the manufacturer’s instructions as described previously^19, 20^. As such, LDL was divided into 7 subfractions and the subfractions of 3 to 7 were grouped into the sdLDL subclass; HDL was divided into 10 subfractions and the subfractions 1 to 3 represented for large HDL. The cholesterol concentration (mg/dl) of each subfraction was subsequently determined.

### Coronary severity assessment

The study patients were subjected to CAG, which was performed using the standard Judkin’s technique with filming of multiple views of each vessel, the results were evaluated by at least two interventional physicians. We assessed coronary severity using Gensini scoring system as described previously^22^. According to the calculated Gensini scores (GS), patients were divided into three subgroups: 0 (normal coronary), 1–23 (low GS) and ≥24 (high GS). In patients who undergone percutaneous transluminal coronary angioplasty (PTCA) or coronary artery bypass grafting (CABG), the angiography-proven coronary severity was measured before the revascularization procedures.

### Statistical analysis

The statistical analyses were performed with SPSS version 19.0 software (SPSS Inc., Chicago, IL, USA). A p-value < 0.05 was considered statistically significant. The values were expressed as mean ± SD (with normally distribution), median (1^st^ to 3^rd^ quantiles, with skewed distribution) for continuous variables and number (percentage) for categorical variables. The differences of clinical and biochemical parameters between groups were examined using analysis of variance, Kruskal-Wallis H test and χ2-tests where appropriate.

Multiple linear and logistic regression analyses with adjustments for confounding factors including age, gender, hypertension, HbA1c, current smoking and hs-CRP were performed to examine the associations between lipids and GS. The lipid couples were modeled in two biomarkers with mutually exclusive composition and consistent significant direction of their associations with high GS. The interaction terms between the two biomarkers were evaluated by the calculated product terms as continuous variables^23^. Taking the couple of TC + apoB for example, it was to multiply the value of TC level in mmol/L by the value of apoB level in g/L as the interaction term between TC and apoB. In addition, the lipid combination categories in each couple were established by 4 groups based on medians of their levels: low/low (less than the medians of both), high/low (greater than or equal to the median of the prior but less than the median of the posterior), low/high (less than the median of the prior but greater than or equal to the median of the posterior) and high/high (greater than or equal to both the medians). Standardized regression coefficients and odds ratios (ORs) with 95% confident intervals (95% CIs) were presented with adjustments for potential confounding factors.

## Results

### Baseline characteristics

Table 1 summarized characteristics of the study patients according to GS status. At baseline, the sample was 62.0% (995) men, with a mean age of 55.5 ± 11.2 years and a mean body mass index (BMI) 25.75 ± 7.06 kg/m^2^. Of the patients, 55.5% were with hypertension, 19.4% were with diabetes mellitus, 77.5% were with dyslipidemia and 32.3% were current smokers. According to GS status, more men were found in low and high GS groups compared to those in normal coronary group (GS = 0). Patients with higher GS had older age, more hypertensions, diabetes, dyslipidemias and smokers.

**Table 1:** Baseline characteristics of patients according to coronary severity.

Variables	GS (0)			GS (1–23)			GS (≥24)			P-value		
	Total	Men	Women	Total	Men	Women	Total	Men	Women	Total	Men	Women
Number	588	276	312	510	340	170	507	379	128	—	—	—
Men,%(n)	46.9(276)	—	—	66.7(340)	—	—	74.8(379)	—	—	**<0.001**	—	—
Age (year)	51.7 ± 12.2	49.5 ± 11.4	53.6 ± 12.5	57.5 ± 10.1	56.3 ± 10.2	60.0 ± 9.5	58.0 ± 9.4	56.4 ± 9.4	62.6 ± 8.8	**<0.001**	**<0.001**	**<0.001**
BMI (kg/m^2^)	25.60 ± 10.71	26.06 ± 3.38	25.18 ± 14.37	25.73 ± 3.29	25.86 ± 3.29	25.46 ± 3.27	25.95 ± 3.73	26.09 ± 3.57	25.53 ± 4.18	0.712	0.640	0.937
Hypertension,%(n)	39.5(232)	41.3(114)	37.8(118)	64.1(327)	62.9(214)	66.5(113)	65.5(332)	63.3(240)	71.9(92)	**<0.001**	**<0.001**	**<0.001**
Diabetes, %(n)	9.5(56)	9.4(26)	9.6(30)	19.4(99)	18.8(64)	20.6(35)	31.0(157)	29.8(113)	34.4(44)	**<0.001**	**<0.001**	**<0.001**
Dyslipidemia,%(n)	75.5(444)	79.7(220)	71.8(224)	75.1(383)	77.6(264)	70.0(119)	82.2(417)	84.2(319)	76.6(98)	0.170	0.078	0.438
Current smoking, %(n)	22.6(133)	43.5(120)	4.2(13)	36.5(186)	52.1(177)	5.3(9)	39.1(198)	48.5(184)	10.9(14)	**<0.001**	0.012	0.084
TG (mmol/L)	1.50(1.08–2.19)	1.59(1.18–2.34)	1.42(1.05–1.97)	1.52(1.10–2.22)	1.49(1.09–2.14)	1.60(1.11–2.38)	1.63(1.22–2.35)	1.62(1.20–2.37)	1.66(1.26–2.30)	**0.015**	0.058	0.007
TC (mmol/L)	4.94 ± 1.14	4.79 ± 1.07	5.07 ± 1.18	4.86 ± 1.04	4.69 ± 0.95	5.19 ± 1.12	4.95 ± 1.08	4.88 ± 1.08	5.13 ± 1.06	0.339	**0.045**	0.520
HDL-C (mmol/L)	1.13 ± 0.36	1.03 ± 0.29	1.22 ± 0.39	1.12 ± 0.36	1.07 ± 0.33	1.23 ± 0.38	1.05 ± 0.30	1.01 ± 0.26	1.19 ± 0.37	**<0.001**	**0.017**	0.673
LDL-C (mmol/L)	3.16 ± 1.07	3.03 ± 0.98	3.27 ± 1.13	3.14 ± 0.95	3.04 ± 0.91	3.35 ± 1.00	3.29 ± 0.97	3.25 ± 0.97	3.39 ± 0.96	**0.037**	**0.002**	0.521
Non-HDL-C (mmol/L)	3.81 ± 1.10	3.76 ± 1.05	3.85 ± 1.15	3.73 ± 1.00	3.62 ± 0.94	3.96 ± 1.08	3.89 ± 1.05	3.88 ± 1.05	3.94 ± 1.04	**0.045**	**0.003**	0.504
ApoA1 (g/L)	1.36 ± 0.31	1.28 ± 0.26	1.43 ± 0.33	1.35 ± 0.32	1.30 ± 0.31	1.44 ± 0.32	1.29 ± 0.27	1.25 ± 0.25	1.40 ± 0.30	**<0.001**	**0.029**	0.569
ApoB (g/L)	1.02 ± 0.19	1.01 ± 0.28	1.03 ± 0.30	1.05 ± 0.28	1.03 ± 0.27	1.11 ± 0.30	1.08 ± 0.27	1.07 ± 0.27	1.10 ± 0.25	**0.004**	**0.01**	0.011
Lp(a) (mg/L)	109.27(56.70–263.20)	88.70(48.32–239.49)	136.03(66.7–289.13)	128.20(57.65–279.42)	121.85(53.36–260.15)	137.18(64.97–312.96)	138.60(66.58–305.5)	129.80(59.50–283.79)	152.05(80.64–349.55)	0.058	**0.039**	0.258
PCSK9 (ng/ml)	226.64(185.78–284.86)	203.96(170.67–266.43)	243.02(199.73–293.01)	226.44(182.24–269.51)	214.18(176.94–259.68)	253.09(205.07–290.33)	234.77(194.79–276.88)	226.96(192.63–266.60)	259.31(205.31–311.45)	**0.049**	**0.002**	0.271
Glucose (mmol/L)	5.28 ± 1.31	5.37 ± 1.62	5.20 ± 0.93	5.64 ± 1.77	5.60 ± 1.88	5.73 ± 1.51	5.91 ± 1.86	5.96 ± 1.94	5.78 ± 1.60	**<0.001**	**<0.001**	**<0.001**
HbA1c(%)	5.78 ± 0.69	5.75 ± 0.80	5.80 ± 0.59	6.03 ± 1.01	6.00 ± 1.00	6.08 ± 1.04	6.39 ± 1.20	6.35 ± 1.21	6.53 ± 1.19	**<0.001**	**<0.001**	**<0.001**
Hs-CRP (mg/L)	1.01(0.58–2.08)	1.00(0.62–2.10)	1.02(0.52–2.05)	1.32(0.69–2.55)	1.15(0.66–2.27)	1.59(0.79–3.03)	1.77(0.83–3.79)	1.66(0.78–3.34)	1.99(0.96–4.65)	**<0.001**	**<0.001**	**<0.001**
Number	401	189	212	374	255	119	397	291	106	—	—	—
ApoC3 (μg/ml)	79.64(52.86–107.85)	77.27(49.50–105.50)	82.55(54.75–109.68)	82.03(58.93–112.87)	78.71(56.54–111.09)	89.36(66.49–113.78)	84.00(58.11–117.79)	81.95(55.13–117.98)	89.28(66.99–117.74)	0.086	0.418	0.658
Number	352	165	187	317	218	99	348	252	96	—	—	—
sdLDL-C (mg/dl)	4.0(2.0–11.0)	4.0(2.0–11.0)	4.0(1.0–13.0)	5.0(2.0–13.0)	5.0(2.0–12.0)	5.0(1.0–16.0)	6.0(2.0–13.0)	6.0(2.0–13.0)	7.0(2.25–14.0)	**0.018**	0.128	0.152
Number	389	184	205	371	253	118	391	286	105	—	—	—
largeHDL-C (mg/dl)	13.0(9.0–17.0)	11.0(8.0–15.0)	13.0(10.0–17.5)	12.0(9.0–17.0)	11.0(8.0–15.7)	14.5(10.0–20.0)	11.0(8.0–15.0)	10.0(8.0–14.0)	14.0(10.0–18.0)	**0.011**	0.077	0.549

Data shown were mean ± SD, media (Q1-Q3) or %(n).

The bold values indicated statistical significance and were bolded to improve the readability of the table.

Abbreviations: GS, Gensini score; BMI, body mass index; TG, triglyceride; TC, total cholesterol; (N) HDL-C, (non) high density lipoprotein cholesterol; LDL-C, low density lipoprotein cholesterol; Apo, apolipoprotein; Lp(a), lipoprotein (a); PCSK9, proprotein convertase subtilisin/kexin type 9; sd, small dense; HbA1c, hemoglobin A1c; Hs-CRP, high-sensitively C-reactive protein.

### Association of each lipid biomarker with coronary severity

As shown in Table 1, the expected direct associations of TG, LDL-C, non-HDL-C, apoB, PCSK9 and sdLDL-C levels with GS status were presented. Also, there were inverse associations of circulating HDL related biomarkers including HDL-C, apoA1, large HDL-C with GS status (all p < 0.05). Furthermore, we performed linear regression analyses (Table 2) with the GS as a quantitative variableand logistic analyses (Table 3) with the GS as a categorical variable (normal coronary, low GS, high GS) to study the predictive values of lipid biomarkers for coronary severity with adjustment for confounding factors.

**Table 2:** Associations of lipid biomarkers with coronary severity using linear regression analysis.

Lipid parameters	Total		Men		Women	
	Coefficients	P-value	Coefficients	P-value	Coefficients	P-value
TG(mmol/L)*	0.019	0.434	0.028	0.393	0.008	0.841
TC(mmol/L)	0.036	0.131	0.065	**0.039**	0.001	0.989
HDL-C(mmol/L)	−0.047	0.059	−0.077	**0.015**	0.009	0.804
LDL-C(mmol/L)	0.075	**0.002**	0.106	**0.001**	0.032	0.402
Non-HDL-C(mmol/L)	0.052	**0.031**	0.091	**0.004**	−0.004	0.922
ApoA1(g/L)	−0.065	**0.008**	−0.073	**0.019**	−0.043	0.264
ApoB(g/L)	0.061	**0.009**	0.092	**0.003**	0.021	0.611
Lp(a)(mg/L)*	0.066	**0.005**	0.066	**0.034**	0.061	0.109
PCSK9 (ng/ml)*	0.054	**0.025**	0.061	**0.045**	0.041	0.297
ApoC3 (μg/ml)*	0.023	0.414	0.017	0.572	0.052	0.199
sdLDL-C (mg/dl)*	0.028	0.389	0.044	0.299	−0.009	0.869
largeHDL-C (mg/dl)*	−0.062	**0.039**	−0.083	**0.031**	−0.016	0.731

Multiple linear regression analyses were performed and standard coefficients were showed with adjustment for age, gender, hypertension, HbA1c, current smoking and hs-CRP.

The bold values indicated statistical significance and were bolded to improve the readability of the table. *Log-transformed data.

Abbreviations as Table 1.

**Table 3:** Associations of lipid biomarkers with coronary severity using logistic regression analysis.

	GS (0)	Total			Men			Women		
		GS (1–23)	GS (≥24)	P for trend	GS (1–23)	GS (≥24)	P for trend	GS (1–23)	GS (≥24)	P for trend
TG(mmol/L)*	Reference	1.02[0.59–1.77]	1.69 [0.96–2.98]	0.095	0.87[0.42–1.77]	1.77[0.88–3.57]	0.084	1.49[0.61–3.65]	1.31[0.46–3.68]	0.680
TC(mmol/L)	Reference	1.03[0.91–1.16]	**1.13[1.00–1.28]**	**0.045**	0.99[0.84–1.17]	**1.17[1.00–1.38]**	**0.025**	1.12[0.94–1.33]	1.04[0.84–1.28]	0.573
HDL-C(mmol/L)	Reference	1.01[0.74–1.29]	**0.62[0.40–0.94]**	**0.043**	0.94[0.53–1.66]	**0.45[0.25–0.82]**	**0.007**	1.19[0.70–1.99]	0.95[0.51–1.76]	0.887
LDL-C(mmol/L)	Reference	1.05[0.92–1.19]	**1.24[1.08–1.42]**	**0.002**	1.05[0.88–1.25]	**1.32[1.11–1.57]**	**0.001**	1.08[0.89–1.30]	1.11[0.89–1.39]	0.413
Non-HDL-C(mmol/L)	Reference	1.02[0.90–1.16]	**1.20[1.05–1.36]**	**0.007**	0.98[0.83–1.17]	**1.26[1.07–1.49]**	**0.002**	1.10[0.92–1.32]	1.05[0.84–1.31]	0.594
ApoA1(g/L)	Reference	0.95[0.82–1.45]	**0.52[0.33–0.83]**	**0.008**	0.90[0.50–1.63]	**0.45[0.24–0.83]**	**0.010**	1.01[0.54–1.89]	0.68[0.33–1.43]	0.348
ApoB(g/L)	Reference	**1.88[1.18–3.00]**	**2.45[1.51–3.97]**	**<0.001**	**2.11[1.11–4.02]**	**3.36[1.77–6.34]**	**<0.001**	1.91[0.97–3.77]	1.44[0.63–3.26]	0.313
Lp(a)(mg/L)*	Reference	1.14[0.88–1.48]	**1.42[1.08–1.87]**	**0.011**	1.15[0.82–1.61]	**1.40[1.00–1.96]**	**0.044**	1.12[0.73–1.71]	1.46[0.89–2.40]	0.152
PCSK9 (ng/ml)*	Reference	1.63[0.58–4.58]	**5.44[1.83–16.21]**	**0.002**	2.96[0.76–11.50]	**10.79[2.74–42.45]**	**<0.001**	0.81[0.16–4.13]	1.37[0.21–9.14]	0.817
ApoC3 (μg/ml)*	Reference	1.69[0.90–3.21]	**2.24[1.07–4.31]**	**0.025**	1.21[0.52–3.03]	1.83[0.78–4.33]	0.247	2.21[0.76–6.42]	2.72[0.83–8.87]	0.098
sdLDL-C (mg/dl)*	Reference	1.26[0.84–1.89]	**1.39[1.00–2.09]**	0.116	1.34[0.78–2.31]	1.52[0.90–2.57]	0.121	1.19[0.63–2.27]	1.22[0.63–2.38]	0.680
largeHDL-C (mg/dl)*	Reference	0.95[0.43–2.12]	**0.36[0.16–0.82]**	**0.013**	0.51[0.17–1.54]	**0.16[0.05–0.49]**	**0.001**	1.64[0.50–5.39]	1.14[0.31–4.19]	0.692

Multiple logistic regression analyses were performed and ORs [95% CIs] were showed with adjustment for age, gender, hypertension, HbA1c, current smoking and hs-CRP.

The bold values indicated statistical significance and were bolded to improve the readability of the table. *Log-transformed data.

Abbreviations as Table 1.

Multiple linear regression analyses suggested that LDL-C, non-HDL-C, apoB, Lp(a) and PCSK9 levels were positively associated with GS, while apoA1 and large HDL-C were negatively associated with GS, respectively (Table 2). When considering only men patients, positive associations with GS were found in TC, LDL-C, non-HDL-C, apoB, Lp(a) and PCSK9, while negative associations with GS were observed in HDL-C, apoA1 and large HDL-C (Table 2). Multiple logistic regression analyses suggested showed that these 12 indices were predictive for high GS (≥24) but not for low GS (1–23) when compared with normal coronary (GS = 0) except for TG (neither) and apoB (both) (Table 3). Data also indicated the above associations in men except for apoC3 (neither) and sdLDL-C (neither) (Table 3). However, no significant association of each biomarker with GS was found in linear and logistic analyses in women (Tables 2 and 3).

### Combined effects of lipid biomarkers on coronary severity

To evaluate the combined effects of biochemical indices on coronary severity, the lipid couples were used. The two biomarkers in a couple should be mainly composed of mutually exclusive components and they both showed significant and consistent associations with GS status in linear and/or logistic models. For example, LDL-C, non-HDL-C and sdLDL-C are all composed of cholesterol and are parts of TC, so we did not analyzed LDL-C, non-HDL-C and sdLDL-C with TC as couples. While, the association directions of TC and apoA1 with high GS were opposite, we also did not included TC with apoA1 as a couple in the analyses. Therefore, 20 eligible couples were analyzed in total population and in men.

As shown in Table 4 regarding the associations of GS as a continuous variable with the lipid interaction terms, significant interactions between lipid biomarkers in couples of TC + Lp(a), HDL-C + apoA1, LDL-C + apoB, LDL-C + Lp(a), LDL-C + PCSK9, LDL-C + apoC3, non-HDL-C + apoB, non-HDL-C + Lp(a), non-HDL-C + PCSK9, apoA1 + large HDL-C, apoB + Lp(a) and apoB + PCSK9 on GS were observed. When considering only men patients, the lipid interactions on GS were found not only in the above couples and also in TC + apoB, TC + PCSK9, non-HDL-C + apoC3 and apoB + apoC3.

**Table 4:** The interactions between lipid biomarkers on coronary severity using linear regression analysis.

Lipid parameters	Total		Men	
	Coefficients	P-value	Coefficients	P-value
TC + apoB	0.046	0.056	0.078	**0.013**
TC + Lp(a)*	0.068	**0.005**	0.086	**0.005**
TC + PCSK9*	0.043	0.074	0.071	**0.023**
TC + apoC3*	0.025	0.4	0.051	0.166
HDL-C + apoA1	−0.065	**0.009**	−0.084	**0.007**
LDL-C + apoB	0.062	**0.009**	0.098	**0.002**
LDL-C + Lp(a)*	0.088	**<0.001**	0.109	**<0.001**
LDL-C + PCSK9*	0.078	**<0.001**	0.109	**<0.001**
LDL-C + apoC3*	0.06	**0.036**	0.093	**0.009**
Non-HDL-C + apoB	0.051	**0.033**	0.088	**0.005**
Non-HDL-C + Lp(a)*	0.077	**<0.001**	0.105	**<0.001**
Non-HDL-C + PCSK9*	0.056	**0.02**	0.095	**0.003**
Non-HDL-C + apoC3*	0.034	0.234	0.072	**0.048**
ApoA1 + largeHDL-C*	−0.066	**0.025**	−0.092	**0.014**
ApoB + Lp(a)*	0.078	**<0.001**	0.096	**0.002**
ApoB + PCSK9*	0.067	**0.005**	0.099	**0.002**
ApoB + apoC3*	0.049	0.089	0.074	**0.043**
ApoB + sdLDL-C*	0.029	0.368	0.054	0.201
PCSK9* + sdLDL-C*	0.031	0.334	0.048	0.249
ApoC3* + sdLDL-C*	0.016	0.621	0.035	0.409

Multiple linear regression analyses were performed and standard coefficients were showed with adjustment for age, gender, hypertension, HbA1c, current smoking and hs-CRP. The interaction terms between the lipid biomarkers in the couples were calculated as the product terms (multiplied the levels of prior lipid by the levels of posterior).

The bold values indicate statistical significance and were bolded to improve the readability of the table. *Log-transformed data.

Abbreviations as Table 1.

In logistic analyses regarding the associations of GS as a categorical variable with the interaction terms (Table 5), data showed significant interactions between lipid biomarkers in couples of apoB + TC, apoB + Lp(a), apoB + PCSK9 on both low GS and high GS. Interactions between lipid biomarkers in all couples except for TC + apoC3, apoB + sdLDL-C, PCSK9 + sdLDL-C and apoC3 + sdLDL-C were found to be related to high GS. These findings suggested the synergism of lipid indices in predicting GS. Furthermore, we found that the ORs of the interactions on GS were increased with elevated GS status. For example, the adjusted ORs of the interactions term in TC + apoB increased from 1.05 [1.00–1.10] at low GS to 1.08 [1.02–1.13] at high GS (p for trend = 0.005). In the subgroup of men patients, data showed that the interactions on GS remained essentially similar.

**Table 5:** The interactions between lipid biomarkers on coronary severity using logistic regression analysis.

	GS (0)	Total			Men		
		GS (1–23)	GS (≥24)	P for trend	GS (1–23)	GS (≥24)	P for trend
TC + apoB	Reference	**1.05[1.00–1.10]**	**1.08[1.02–1.13]**	**0.005**	1.04[0.97–1.12]	**1.11[1.04–1.19]**	<**0.001**
TC + Lp(a)*	Reference	1.02[0.98–1.06]	**1.06[1.02–1.10]**	**0.002**	1.01[0.96–1.07]	**1.07[1.02–1.12]**	**0.003**
TC + PCSK9*	Reference	1.01[0.97–1.06]	**1.06[1.01–1.11]**	**0.018**	1.00[0.94–1.07]	**1.08[1.02–1.15]**	**0.006**
TC + apoC3*	Reference	1.01[0.95–1.03]	1.02[0.97–1.08]	0.338	0.95[0.88–1.03]	1.03[0.97–1.11]	0.157
HDL-C + apoA1	Reference	1.00[0.86–1.17]	**0.76[0.63–0.91]**	**0.008**	0.97[0.77–1.24]	**0.68[0.53–0.89]**	**0.004**
LDL-C + apoB	Reference	1.05[0.99–1.12]	**1.11[1.04–1.18]**	**0.002**	1.07[0.97–1.17]	**1.17[1.07–1.27]**	**<0.001**
LDL-C + Lp(a)*	Reference	1.02[0.98–1.07]	**1.09[1.04–1.14]**	**<0.001**	1.02[0.96–1.09]	**1.10[1.04–1.17]**	**<0.001**
LDL-C + PCSK9*	Reference	1.02[0.97–1.08]	**1.10[1.04–1.16]**	**<0.001**	1.03[0.96–1.11]	**1.13[1.05–1.22]**	**<0.001**
LDL-C + apoC3*	Reference	1.01[0.94–1.05]	**1.06[1.01–1.14]**	**0.041**	1.01[0.93–1.08]	**1.10[1.00–1.21]**	**0.011**
Non-HDL-C + apoB	Reference	1.05[0.99–1.11]	**1.09[1.03–1.16]**	**0.003**	1.04[0.96–1.13]	**1.13[1.05–1.22]**	**<0.001**
Non-HDL-C + Lp(a)*	Reference	1.02[0.98–1.07]	**1.08[1.04–1.13]**	**<0.001**	1.01[0.95–1.07]	**1.10[1.04–1.16]**	**<0.001**
Non-HDL-C + PCSK9*	Reference	1.01[0.96–1.06]	**1.08[1.03–1.14]**	**0.003**	1.00[0.93–1.07]	**1.11[1.04–1.19]**	**<0.001**
Non-HDL-C + apoC3*	Reference	1.01[0.92–1.03]	**1.04[1.00–1.10]**	**0.047**	0.95[0.87–1.03]	**1.06[1.00–1.15]**	**0.042**
ApoA1 + largeHDL-C*	Reference	0.97[0.73–1.30]	**0.69[0.51–0.94]**	**0.019**	0.78[0.52–1.17]	**0.50[0.33–0.77]**	**0.002**
ApoB + Lp(a)*	Reference	**1.23[1.05–1.43]**	**1.37[1.17–1.61]**	**<0.001**	**1.26[1.02–1.55]**	**1.47[1.19–1.80]**	**<0.001**
ApoB + PCSK9*	Reference	**1.28[1.06–1.54]**	**1.46[1.21–1.77]**	**<0.001**	**1.40[1.06–1.79]**	**1.71[1.33–2.21]**	**<0.001**
ApoB + apoC3*	Reference	1.10[0.98–1.36]	**1.25[1.03–1.58]**	**0.027**	1.12[0.87–1.54]	**1.44[1.06–1.95]**	**0.009**
ApoB + sdLDL-C*	Reference	1.17[0.89–1.54]	1.22[0.93–1.60]	0.148	1.22[0.83–1.78]	1.31[0.91–1.89]	0.134
PCSK9* + sdLDL-C*	Reference	1.09[0.92–1.30]	1.16[0.98–1.37]	0.095	1.13[0.90–1.42]	1.21[0.97–1.51]	0.087
ApoC3* + sdLDL-C*	Reference	1.05[0.87–1.27]	1.10[0.91–1.33]	0.297	1.09[0.85–1.40]	1.17[0.92–1.48]	0.193

Multiple logistic regression analyses were performed and ORs [95% CIs] were showed with adjustment for age, gender, hypertension, HbA1c, current smoking and hs-CRP. The interaction terms as Table 4.

The bold values indicated statistical significance and were bolded to improve the readability of the table. *Log-transformed data.

Abbreviations as Table 1.

In an alternative analysis, which evaluated lipid biomarkers in the couple in combination with a categorical trait, we investigated the predictive values of different lipid combinations for GS (Table 6). The ORs of the discordant categories (low/high and high/low) for low GS were found to be not significant in the adjusted models statistically. In contrast, the ORs of the concordant categories with both at high levels (high/high) for low GS were significantly higher than those for normal coronary in couples of TC + apoB, LDL-C + apoB, non-HDL-C + apoB, apoB + Lp(a), apoB + PCSK9 and apoB + apoC3 (all p and p for trend < 0.05). The ORs were 1.39 [1.04–1.87], 1.40 [1.04–1.88]. 1.35 [1.01–1.80], 1.59 [1.12–2.27], 1.54 [1.10–2.17] and 1.65 [1.02–2.66], respectively. Thus, these results suggested that a high apoB conferred a greater risk for low GS when given in combination with elevations in TC, etc. Except for TG, other 11 indices were significantly predictive for high GS (Table 3). However, the ORs of the discordant categories (low/high and high/low) for high GS were found to be statistically significant in only couples of LDL-C + PCSK9 and non-HDL-C + PCSK9. The ORs of high LDL-C/low PCSK9 as well as low LDL-C/high PCSK9 and high non-HDL-C/low PCSK9 as well as low non-HDL-C/high PCSK9 were 1.70 [1.16–2.49], 1.63 [1.11–2.41],1.88 [1.28–2.76], 1.73 [1.17–2.57] for high GS, respectively. Interestingly, high PCSK9 was significantly predictive for high GS even if TC was at low levels (1.63 [1.11–2.40]) but the discordant category of low PCSK9/high TC was not (1.42 [0.97–2.09]). Both high apoB and high sdLDL-C in combination with low PCSK9 were predictive for high GS (1.48 [1.01–2.17], 1.72 [1.10–2.68]) but high PCSK9 in combination with either low apoB or low sdLDL-C was not (1.39 [0.95–2.05], 1.44 [0.88–2.21]). In addition, high non-HDL-C/low Lp(a), low PCSK9/high sdLDL-C also showed significant values (1.50 [1.03–2.19], 1.70 [1.04–2.78]). Importantly, the concordant categories of adverse lipid levels in combination in all 20 couples showed the highest predictive values for high GS (all p and p for trend <0.05), suggesting that the adverse lipid combinations were associated with increased risk of coronary severity.

**Table 6:** Associations of lipid combinations with coronary severity using Logistic regression analysis.

	GS (0)	GS (1–23)				GS (≥24)			
		1	2	3	P for trend	1	2	3	P for trend
Total									
TC + apoB	Reference	0.87[0.56–1.35]	1.15[0.74–1.79]	**1.39[1.04–1.87]**	**0.018**	0.82[0.51–1.32]	1.24[0.79–1.95]	**1.57[1.16–2.13]**	**0.002**
TC + Lp(a)	Reference	1.03[0.71–1.47]	0.99[0.69–1.43]	1.39[0.99–1.42]	0.084	1.27[0.87–1.86]	1.24[0.85–1.81]	**1.61[1.11–2.32]**	**0.017**
TC + PCSK9	Reference	1.25[0.87–1.80]	1.24[0.85–1.79]	1.38[0.98–1.94]	0.082	1.42[0.97–2.09]	**1.63[1.11–2.40]**	**1.78[1.24–2.56]**	**0.001**
TC + apoC3	Reference	0.84[0.54–1.31]	0.98[0.63–1.52]	1.53[0.91–1.52]	0.102	1.08[0.71–1.45]	0.96[0.60–1.49]	**1.82[1.07–3.11]**	**0.048**
HDL-C + apoA1	Reference	0.92[0.60–1.42]	0.97[0.63–1.49]	0.94[0.71–1.26]	0.728	0.79[0.52–1.23]	0.94[0.61–1.44]	**0.64[0.47–0.86]**	**0.005**
LDL-C + apoB	Reference	1.17[0.76–1.81]	1.52[0.99–2.33]	**1.40[1.04–1.88]**	**0.017**	1.38[0.88–2.18]	1.43[0.91–2.26]	**1.75[1.29–2.37]**	**<0.001**
LDL-C + Lp(a)	Reference	0.97[0.67–1.39]	0.93[0.65–1.34]	1.39[0.99–1.95]	0.086	1.38[0.94–2.01]	1.12[0.76–1.64]	**1.83[1.28–2.62]**	**0.004**
LDL-C + PCSK9	Reference	1.16[0.81–1.67]	1.13[0.79–1.63]	1.39[0.99–1.95]	0.077	**1.70[1.16–2.49]**	**1.63[1.11–2.41]**	**2.07[1.44–2.98]**	**<0.001**
LDL-C + apoC3	Reference	1.18[0.76–1.82]	1.37[0.89–2.11]	1.40[0.88–2.22]	0.117	1.45[0.93–2.24]	1.25[0.81–1.77]	**1.74[1.09–2.78]**	**0.044**
Non-HDL-C + apoB	Reference	0.68[0.43–1.07]	1.01[0.65–1.56]	**1.35[1.01–1.80]**	**0.030**	0.97[0.61–1.53]	0.83[0.51–1.34]	**1.75[1.29–2.37]**	**<0.001**
Non-HDL-C + Lp(a)	Reference	1.03[0.72–1.48]	1.06[0.74–1.51]	1.37[0.96–1.95]	0.099	**1.50[1.03–2.19]**	1.18[0.80–1.73]	**1.97[1.36–2.86]**	**0.002**
Non-HDL-C + PCSK9	Reference	1.10[0.77–1.59]	1.13[0.78–1.63]	1.31[0.94–1.85]	0.13	**1.88[1.28–2.76]**	**1.73[1.17–2.57]**	**2.18[1.51–3.15]**	**<0.001**
Non-HDL-C + apoC3	Reference	1.02[0.65–1.60]	1.18[0.76–1.85]	1.59[0.93–2.71]	0.076	1.46[0.92–2.33]	1.08[0.67–1.74]	**2.46[1.43–4.25]**	**0.018**
ApoA1 + largeHDL-C	Reference	1.07[0.68–1.69]	0.67[0.41–1.09]	0.94[0.64–1.38]	0.514	1.13[0.73–1.75]	0.66[0.42–1.06]	**0.53[0.36–0.78]**	**0.001**
ApoB + Lp(a)	Reference	1.06[0.74–1.52]	0.90[0.62–1.29]	**1.59[1.12–2.27]**	**0.031**	1.36[0.93–1.98]	1.10[0.75–1.60]	**1.93[1.33–2.80]**	**0.003**
ApoB + PCSK9	Reference	1.16[0.81–1.67]	0.98[0.68–1.42]	**1.54[1.10–2.17]**	**0.029**	**1.48[1.01–2.17]**	1.39[0.95–2.05]	**2.07[1.45–2.98]**	**<0.001**
ApoB + apoC3	Reference	1.36[0.88–2.11]	1.41[0.91–2.16]	**1.65[1.02–2.66]**	**0.047**	**1.72[1.10–2.68]**	1.44[0.88–2.21]	**1.86[1.14–3.04]**	**0.039**
ApoB + sdLDL-C	Reference	1.17[0.71–1.92]	1.08[0.67–1.76]	1.20[0.80–1.78]	0.408	1.48[0.89–2.46]	1.45[0.88–2.37]	**1.93[1.29–2.89]**	**0.002**
PCSK9 + sdLDL-C	Reference	0.81[0.52–1.27]	1.02[0.63–1.64]	1.01[0.64–1.59]	0.773	1.23[0.77–1.96]	**1.70[1.04–2.78]**	**1.77[1.10–2.84]**	**0.008**
ApoC3 + sdLDL-C	Reference	1.00[0.63–1.58]	0.98[0.62–1.55]	1.40[0.78–2.51]	0.406	1.19[0.73–1.92]	1.53[0.95–2.45]	**2.21[1.22–4.00]**	**0.005**
Men									
TC + apoB	Reference	0.75[0.41–1.36]	1.16[0.66–2.02]	1.28[0.87–1.89]	0.169	0.69[0.37–1.28]	1.26[0.72–2.21]	**1.61[1.01–1.13]**	**0.010**
TC + Lp(a)	Reference	0.94[0.59–1.50]	1.16[0.74–1.84]	1.46[0.92–2.31]	0.095	1.22[0.77–1.93]	1.31[0.83–2.08]	**1.72[1.08–2.73]**	**0.023**
TC + PCSK9	Reference	1.08[0.69–1.69]	1.24[0.76–2.01]	1.27[0.81–2.00]	0.245	1.29[0.82–2.03]	**1.72[1.06–2.79]**	**1.87[1.20–2.94]**	**0.003**
TC + apoC3	Reference	0.62[0.34–1.13]	0.83[0.47–1.46]	1.51[0.74–3.10]	0.239	0.85[0.47–1.52]	0.72[0.41–1.29]	1.83[0.90–3.71]	0.263
HDL-C + apoA1	Reference	0.89[0.43–1.51]	0.98[0.70–1.62]	0.92[0.66–1.31]	0.841	0.74[0.37–1.21]	0.82[0.49–1.33]	**0.56[0.31–0.94]**	**0.035**
LDL-C + apoB	Reference	1.00[0.57–1.77]	1.42[0.82–2.45]	1.28[0.87–1.89]	0.159	1.25[0.71–2.20]	1.25[0.71–2.21]	**1.85[1.26–2.73]**	**0.002**
LDL-C + Lp(a)	Reference	0.96[0.60–1.52]	1.18[0.74–1.87]	1.44[0.91–2.27]	0.097	1.47[0.93–2.32]	1.24[0.77–2.00]	**2.06[1.31–3.25]**	**0.005**
LDL-C + PCSK9	Reference	1.01[0.64–1.57]	1.12[0.70–1.82]	1.31[0.83–2.05]	0.239	**1.59[1.01–2.49]**	**1.63[1.01–2.66]**	**2.30[1.46–3.62]**	**<0.001**
LDL-C + apoC3	Reference	1.00[0.56–1.80]	1.24[0.71–2.15]	1.41[0.76–2.61]	0.211	1.41[0.80–2.51]	1.00[0.56–1.74]	**1.86[1.01–3.41]**	0.148
Non-HDL-C + apoB	Reference	0.64[0.36–1.12]	0.93[0.53–1.65]	1.28[0.87–1.90]	0.172	0.85[0.49–1.48]	0.68[0.37–1.26]	**1.85[1.25–2.73]**	**0.003**
Non-HDL-C + Lp(a)	Reference	0.93[0.59–1.48]	1.14[0.72–1.83]	1.45[0.91–2.32]	0.095	1.44[0.91–2.28]	1.18[0.73–1.91]	**2.18[1.36–3.49]**	**0.004**
Non-HDL-C + PCSK9	Reference	1.09[0.70–1.71]	1.28[0.78–2.11]	1.26[0.80–1.98]	0.256	**1.84[1.17–1.17]**	**1.94[1.17–3.22]**	**2.36[1.50–3.72]**	**<0.001**
Non-HDL-C + apoC3	Reference	0.77[0.41–1.45]	0.94[0.51–1.71]	1.77[0.83–3.78]	0.129	1.15[0.62–2.13]	0.80[0.43–1.49]	**2.72[1.28–5.76]**	0.059
ApoA1 + largeHDL-C	Reference	1.11[0.61–2.00]	**0.42[0.22–0.81]**	0.77[0.46–1.29]	0.137	1.41[0.81–2.49]	0.55[0.30–1.02]	**0.48[0.28–0.82]**	**0.004**
ApoB + Lp(a)	Reference	1.02[0.64–1.61]	1.02[0.64–1.62]	**1.77[1.10–2.84]**	**0.034**	1.41[0.89–2.23]	1.19[0.74–1.89]	**2.21[1.37–3.55]**	**0.004**
ApoB + PCSK9	Reference	1.16[0.75–1.82]	1.05[0.65–1.71]	1.55[0.98–2.46]	0.101	**1.58[1.01–2.48]**	**1.60[1.00–2.59]**	**2.36[1.49–3.75]**	**<0.001**
ApoB + apoC3	Reference	1.40[0.78–2.54]	1.43[0.82–2.50]	1.83[0.97–3.48]	0.081	**1.82[1.02–3.24]**	1.25[0.72–2.20]	**2.05[1.09–3.86]**	0.117
ApoB + sdLDL-C	Reference	1.22[0.62–2.38]	1.01[0.55–1.84]	1.30[0.77–2.22]	0.407	1.66[0.86–3.21]	1.31[0.72–2.38]	**2.11[1.25–3.56]**	**0.009**
PCSK9 + sdLDL-C	Reference	0.87[0.49–1.55]	1.16[0.65–2.06]	0.99[0.54–1.79]	0.825	1.35[0.76–2.41]	1.73[0.97–3.09]	**1.78[1.00–3.22]**	**0.033**
ApoC3 + sdLDL-C	Reference	0.93[0.50–1.72]	0.94[0.49–1.79]	1.46[0.66–3.23]	0.418	0.89[0.48–1.65]	1.26[0.67–2.38]	1.85[0.85–4.05]	0.055

Multiple ordinal logistic regression analyses were performed, ORs [95% CIs] were showed with adjustment for age, gender, hypertension, HbA1c, current smoking and hs-CRP.

GS status was the dependent variable with the group of GS = 0 as reference status. Lipid categories of the two biomarkers were the independent variable, and 4 groups were included based on the medians of their levels: low/low (less than the medians of both), high/low (greater than or equal to the median of the prior but less than the median of the posterior), low/high (less than the median of the prior but greater than or equal to the median of the posterior) and high/high (greater than or equal to both the medians). The OR of low/low was 1 and not shown in the Table.

The medians of their levels were 4.77 mmol/L (TC), 1.04 mmol/L (HDL-C), 3.07 mmol/L (LDL-C), 3.67 mmol/L (non-HDL-C), 1.30 g/L (apoA1), 1.01 g/L (apoB), 123.34 mg/L [Lp(a)], 228.48 ng/ml (PCSK9), 82.23 μg/ml (apoC3), 12.0 mg/dl (large HDL-C), 5.0 mg/dl (sdLDL-C) in total population and 4.66 mmol/L (TC), 0.98 mmol/L (HDL-C), 3.04 mmol/L (LDL-C), 3.66 mmol/L (non-HDL-C), 1.26 g/L (apoA1), 1.01 g/L (apoB), 112.48 mg/L [Lp(a)], 218.75 ng/ml (PCSK9), 78.43 μg/ml (apoC3), 11.0 mg/dl (large HDL-C), 5.0 mg/dl (sdLDL-C) in men.

The bold values indicated statistical significance and were bolded to improve the readability of the table.

Abbreviations as Table 1.

## Discussion

The present study explored two important patterns of associations of circulating lipid biomarkers with coronary severity in a cohort of non-lipid-lowering-drug-treated patients undergoing the first CAG. The main findings of this study were as follows. Firstly, we enrolled the current available 12 lipid indices and evaluated their respective value in predicting GS. In multiple linear regression analyses with GS as a continuous variable, levels of LDL-C, non-HDL-C, apoB, Lp(a) and PCSK9 showed the positive predictive values for GS, while apoA1 and large HDL-C showed the negative values. In multiple logistic regression analyses with GS as a categorized variable, these 12 indices were predictive for high GS but not for low GS except for TG (neither) and apoB (both). Further gender stratification analyses showed similar associations between lipids and GS in men but not in women. Secondly, 20 eligible lipid couples were built to clarify the combined patterns of two lipid biomarkers in predicting GS. We found that the two indices in a couple acted synergistically in predicting GS and the interactions between the two were increased as the GS status elevated. Importantly, the concordant categories of adverse lipid levels in combination in all 20 couples showed the highest predictive values for high GS when the combined patterns were established by 4 groups based on medians of their levels: low/low, high/low, low/high and high/high. To the best of our knowledge, this was the first study to address directly the associations of 12 established and novel lipid biomarkers, alone and in combination, with coronary severity in a large cohort of non-lipid-lowering-drug treated patients. The number of the enrolled indices, the relative comprehensive analyses and the study population with the untreated or original lipid levels might be the highlights of the present study.

Dyslipidemia plays an essential role in the initiation and progression of CAD and its clinical consequences^6, 7^. Increasing evidences indicate the relationships of abnormal lipid and lipoprotein biomarkers with the development of CAD^5, 9^. Traditionally, lipid and lipoprotein profile includes information on TG, TC, HDL-C, LDL-C, apoA1, apoB and Lp(a). Nevertheless, some of them remain to be measured infrequently in routine clinical practice. For example, despite the recognition of Lp(a) as an independent risk factor of CAD, irrespective of other coexisting lipids, physicians’ knowledge on Lp(a) is limited^10^. The lack of clear recommendation for the cut-off value or poor therapeutic options for patients with high levels of this biomarker may be the reasons. Taking the above into account, a recent study from Afshar *et al.* is of special interest^11^. They reported that in ACS patients (<55 years), high Lp(a) was strongly associated with high LDL-C levels and Lp(a) conferred a greater risk for premature ACS when LDL-C was elevated, highlighting the physiological link between Lp(a) and LDL-C, especially the potential importance of LDL-C in Lp(a) >50 mg/dL patients. Thus, although we know so much for the traditional lipid biomarkers, further studies are still required.

Furthermore, clinical interest has focused on emerging lipid parameters such as PCSK9 (target specific proteins for LDL-C)^17^, apoC3^18^, HDL and LDL particle^15^ in relation to cardiovascular risk. Direct comparisons of the predictive values for coronary severity in these parameters have been rare yet. Moreover, it is controversial whether any of these biomarkers have independent prognostic value^24^. The present study confirmed significant associations between these novel biomarkers and coronary severity. The results including 12 current available indices reflected at least in part the notion that it was lipid or lipoprotein abnormalities other than traditional lipid measurements or LDL-C that swept up the lipid-related information on coronary severity. Also, dyslipidemia is far more than current classification such as hypertriglyceridemia, hypercholesterolemia or hypo HDL cholesterolemia. For a long time until now, statin therapy stands as a bulwark in the frontier of therapeutic strategies for patients with dyslipidemia and/or CAD and the chronicled successes are obtained from the use of statins^25–27^. However, a significant number of individuals with hypercholesterolemia do not achieve the optimal levels of LDL-C^14^. Analyses of clinical trial data also reveal significant residual cardiovascular risk in all patients treated with statins even in the setting of optimal LDL-C reduction^14^. The fact might be explained by lack of efficacy or adverse effects, highlighting the need to retool cardiovascular risk reduction algorithms beyond focusing on LDL-C levels and/or the use of statins. Of note, studies have confirmed consistently that statin treatment increases the expression of PCSK9 in both normolipidemic and dyslipidemic subjects regarding that low intracellular cholesterol levels control gene expression of both LDLR and PCSK9 via nuclear translocation of sterol regulatory element binding protein-2 (SREBP-2)^28^. SREBP–2-mediated LDLR expression increases hepatic LDL-C uptake while SREBP-2 induces expression of PCSK9, which enhances hepatic LDLR degradation, thus preventing excessive cholesterol uptake in order to preserve cholesterol homeostasis^28^. Accordingly, missense mutations and loss-of function mutations in PCSK9 gene are associated with increased statin response and hypocholesterolemia, pointing to the potential benefit of PCSK9 inhibition and its potentially additive effect in combination with statins^29, 30^. Our data also showed the significant value of PCSK9 in predicting coronary severity. Therefore, additional information on emerging lipid biomarkers is warranted in clinical practice.

Importantly, we investigated the combined effects of lipid biomarkers, traditional or new emerging, and found that combined abnormalities were associated with increased risk of coronary severity. For low GS, the high levels of lipid biomarkers absolute alone were not significantly predictive but the combined applications for high apoB with TC, LDL-C, non-HDL-C, Lp(a), PCSK9 and apoC3 manifested the significance. The groups with high/high had the highest risk for high GS, while the majority of the discordant groups with low/high or high/low were not valuable in predicting severity. The combinations of lipid-associated atherogenic biomarkers might help identify patients who were at high cardiovascular risk or warranted aggressive treatment for the dyslipidemia complex^11, 31–33^.

Finally, when gender stratification analyses were performed, we found that none of these lipid indices were correlated with GS in women. The associations of these biomarkers with GS were only significant for men in our study patients. These results were in contrast to some prior studies including the Framingham Study^34, 35^, but in agreement with other studies^36–38^. Several concerns might be considered. First, the women that were included in our study had an adverse lipid profile compared with men, while the presence of CAD was considerably lower [48.9% (298/610) vs. 72.3% (719/995)] and the proportion of CAD was decreased with elevated GS status (53.1% at normal coronary vs. 33.3% at low GS vs. 25.2% at high GS, p < 0.001). These results were largely accordance with previous studies, supporting the notion of gender difference in the development of CAD^38, 39^. Second, men were more likely to have unhealthy lifestyles and unfavorable psychosocial factors compared with women and these risk factors might accelerate the effect of dyslipidemia on CAD development^38^. In the present study, men were more likely to smoke compared with women. In addition, our study and many other studies on CAD have included mostly men, the knowledge on the associations in women might be in need of further specific evaluation^37^.

Therefore, the implications of the present study should be emphasized. Our fundamental understanding on the role of dyslipidemia in CAD mainly comes from LDL-C. The present study, however, suggested that other lipid indices, alone or in combination, carried important information in severity evaluation of CAD. Although the attributable risk or synergistic effect had been recognized, they still have much to learn. Furthermore, our results addressed that the dyslipidemia management in CAD patients required a comprehensive perspective, lifestyle modification and/or pharmacologic therapy aimed at improving the lipid profile rather than an individual lipid parameter might provide more cardiovascular benefits in clinical practice. In addition, the gender dichotomy of lipid-related risk might underlie the increased propensity to CAD in men. On the other hand, the findings of the present study should be interpreted in light of limitation. First, many other risk factors, such as LDL or HDL particle size, number, VLDL, IDL, etc were not included in the analyses. Second, the study was with cross-sectional nature, prospective study with a follow-up for the development of coronary severity might strengthen the results. Third, the lack of the associations between lipids and coronary severity in women might need a larger sample to confirm. Finally, we used GS, a surrogate marker of coronary severity, as the outcome. However, strong association has been shown between GS and subsequent CAD prognosis and GS is recognized as preferred scoring system with predominantly anatomy and angiographic findings evaluated^40, 41^. The present study regarding the associations of lipid biomarkers with coronary severity might provide novel sight to future disease improvement.

In conclusion, the present study, we addressed simultaneously 12 lipid or lipoprotein biomarkers that had been recognized to be associated with an increased/decreased risk for the development of CAD. Our data showed that (1) new emerging lipid biomarkers including PCSK9, apoC3, sdLDL and large HDL, consisting with the traditional lipids and lipoproteins, were associated significantly with coronary severity; (2) significant interactions between lipid biomarkers on coronary severity were observed and the predictive values of the interactions for severity were increased with elevated GS status; (3) adverse lipid combinations increased the values in predicting coronary severity: for low severity, high apoB in combination with high TC, LDL-C, non-HDL-C, Lp(a), PCSK9 and apoC3 showed the greatest risks compared to other 3 lipid categories; while for high severity, the adverse lipid combinations showed the highest values in all 20 couples, suggesting that the collective assessment of lipid biomarkers might facilitate risk identification and clinical management. Larger prospective studies are needed to confirm our findings.
